# Effect of selenium nanoparticles on the quality and fertility of short-term preserved boar semen

**DOI:** 10.3389/fvets.2023.1333841

**Published:** 2024-01-23

**Authors:** Dipan Rudra Paul, Dibyajyoti Talukdar, Fazal Ali Ahmed, K. Lalrintluanga, Girin Kalita, T. C. Tolenkhomba, Himsikha Chakravarty, Rahul Katiyar, Gautam Khargharia, Sourabh Deori

**Affiliations:** ^1^College of Veterinary Sciences & A.H, Central Agricultural University, Aizawl, Mizoram, India; ^2^Division of Animal and Fisheries Sciences, ICAR Research Complex for NEH Region, Umiam, Meghalaya, India

**Keywords:** selenium nanoparticles, boar semen, BTS extender, liquid preservation, antioxidants

## Abstract

This study was carried out to investigate the effect of different concentrations of selenium nanoparticles (Se-NPs) in the Beltsville Thawing Solution (BTS) extender on the semen quality and fertility of Hampshire crossbred pigs. For the study, semen was collected from four boars (10 ejaculates/boar) by the gloved hand method. Each ejaculate was extended @ 1:2 with the BTS extender and split into four aliquots. The control (C) samples were without the supplementation of Se-NPs, whereas the other three were supplemented with 0.5 (T1), 1 (T2), and 2 μg ml^−1^ of Se-NPs (T3) and stored at 15°C in a BOD incubator. Extended semen was evaluated at 0 (immediately after dilution), 24, 48, 72, and 96 h of storage for sperm motility, live sperm, plasma membrane integrity, acrosome integrity, DNA integrity, and mitochondrial membrane potential (MMP). The mean percentage of sperm motility, live sperm, and sperm with intact plasma membrane and acrosome, and MMPs were significantly (*p* < 0.01) higher in all treated groups in comparison to control at 24, 48, 72, and 96 h of storage. Sperm with intact DNA in all treated groups increased significantly at 48 (*p* < 0.05), and 72 and 96 (*p* < 0.01) h of storage in comparison to the control group. The concentration of 1 μg ml^−1^ of Se-NPs was found to be the best among other concentrations. In each group, 10 sows were artificially inseminated with the liquid semen preserved for 72 h at 15°C. Supplementation of 1 μg ml^−1^ of Se-NPs yielded the highest conception rate in comparison to other groups. In conclusion, supplementation of 1 μg ml^−1^ of Se-NPs in the BTS extender resulted in the best semen quality and conception rate during the short-time liquid preservation of boar semen.

## 1 Introduction

Artificial insemination is frequently carried out in swine farms all over the world with extended liquid storage of semen to maintain the sperm fertilizing ability (1). Semen extenders allow the extraction of numerous doses from a single ejaculate (2); additionally, they allow the maintenance of sperm viability at 16–18°C for days (3). Commercial extenders are categorized as short, medium, and long-term depending on their capacity to keep sperm for 1 to 2, 3 to 4, or 7 to 10 days after collection (4). However, due to the fact that cell metabolism does not slow down or cease on cooling (5°C) or freezing (−196°C), which creates favorable conditions for microbes to damage the quality of the sample, their storage capacity is restricted (5, 6). Modification of the sperm collection protocol can reduce contamination in raw sperm by 49.85% in bacteria and by 9.67% in fungi (7). The bacteria and filamentous fungi in boar semen could be controlled by sanitary procedures and by using commercial extenders (8). They also reported that the fertility of AI in sows with lower microspermia generated better results compared to the high one. Boar semen, kept in liquid condition that day or held at 15–20°C for 1–5 days, is used in almost 99% of artificial inseminations that are performed globally (1). The sperm quality deteriorates gradually on storage for prolonged periods due to oxidative stress (9, 10). The majority of the cells neutralize the toxic effects of various reactive oxygen species (ROS); however, the antioxidant system in the sperm cell is much lower as compared to other cells and more susceptible to oxidative stress (11). Mammals, fish, and birds have an abundance of unsaturated fatty acids and phospholipids in their sperm plasma membrane. Increased levels of unsaturated fatty acids with multiple bonds make the sperm cell sensitive to lipid peroxidation, which has a positive correlation with male infertility (12). ROS are generated when fatty acids are oxidized. Under normal circumstances, these radicals are required for several actions and physiological processes in the sperm; however, an excess amount of ROS generation may cause a decrease in membrane fluidity, DNA bridging, damaged proteins, and finally lowered sperm motility and fertility (13). Selenium is a potent natural antioxidant that has the capacity to stop spermatozoa from oxidizing. Recently, nanoparticles emerged as promising alternatives that suppressed toxicity but maintained the positive effects of selenium on an organism (14). Urbankova et al. studied the effects of sub-lethal doses of Se-NPs on the health status of rats and suggested that short-term Se-NP supplementation can be safe and beneficial in cases of Se deficiency or specific treatment (15). Several studies reported the beneficial effect of supplementing semen extenders with antioxidants on semen quality during processing and cryopreservation (16). Researchers also reported a strong favorable relationship between sperm quality and selenium levels in the seminal plasma.

The enzyme GSH-PX uses selenium as one of its constituents and protects against peroxidative injury to cell membranes and other organelles that constitute lipids (17). Over the past two decades, a growing corpus of research has examined the effects of NPs on semen characteristics. NPs with a diameter of less than 100 nm can be applied to different reproductive biology techniques due to their physiochemical characteristics (18). The antioxidant properties of certain NPs are the most intriguing aspect of their usage in maintaining sperm cell activity during preservation (19). Horkey et al. reported that the oral supplementation of selenium nanoparticles has not shown an improving effect on sperm quality. However, this could be considered a safe alternative to inorganic selenium, as well as having the potential to enhance the antioxidant properties of the semen of boars (20).

The bioactive abilities of selenium in nanoforms obtained by the application of nanotechnology can be exploited in reproduction, growth, cell freezing, digesting, and antibacterial activities. Additionally, a number of clinical and experimental studies have looked at the impact of Se-NP administration on the quality of the semen in goat bucks, rats, and roosters grown *in vitro*. However, there is no study to optimize the level of supplementation of Se-NPs in the extender for improving boar semen quality. As selenium has antioxidant capabilities, it was hypothesized that this would protect the sperm population from oxidative damage and improve semen quality and fertility.

## 2 Materials and methods

The study was conducted at the Division of Animal and Fisheries Sciences, ICAR Research Complex for the North Eastern Hill Region, Umiam, Meghalaya, India. Four mature, healthy Hampshire crossbred boars, ranging in age from 2.5 to 3 years with normal reproductive characteristics, were used for the experiment. The selenium nanoparticles (Se-NPs) were commercially purchased from Sai-Biotech, India (catalog number 282492). The size of the selenium nanoparticles was 80 nm. The research study was duly approved by the Institution Animal Ethics Committee of the College of Veterinary Science and Animal Husbandry, Central Agricultural University, Aizawl, India.

### 2.1 Semen collection and processing

Forty semen ejaculates were collected from trained crossbred Hampshire boars (n = 4, 10 ejaculates from each boar) by the gloved hand method using a dummy by the same technician following a twice-weekly schedule. The boars were aged between 14 and 18 months and managed under similar conditions. Following ejaculation, the gel fraction was separated by filtering through gauze using a Buchner funnel. Fresh semen samples were evaluated for volume by using a measuring cylinder, sperm motility by direct observation under a microscope, and sperm concentration by a Neubauer counting chamber. Samples exhibiting mass activity ≥3+, sperm motility ≥70%, viability ≥75%, and sperm concentration ≥ 100 million/ml were processed further for the experiment.

Each ejaculate was extended with a BTS extender @ 1:2 (v/v) ratio to achieve a final concentration of at least 50 million spermatozoa per ml of semen and divided into four aliquots. Each aliquot was supplemented with 0 (control), 0.5 (T1), 1 (T2), and 2 (T3) μg ml^−1^ Se-NPs and stored at 15°C in a BOD incubator. At 0, 24, 48, 72, and 96 h of preservation, the stored semen samples were evaluated for sperm motility by the conventional method, live sperm count by eosin-nigrosin staining, plasma membrane integrity by the hypo-osmotic swelling test (HOST), acrosomal integrity by Giemsa staining, DNA integrity by acridine orange staining, and MMP using JC-1 dye.

### 2.2 Total sperm motility

A drop of semen that had been pre-warmed to 37°C was put on a clean glass slide, covered with a cover slip, and examined under 40x magnification under a microscope. The proportion of motile spermatozoa was recorded.

### 2.3 Live sperm

The live sperm percentage was determined by the eosin–nigrosin staining technique (21). The straining solution (10 μL) was mixed with 10 μL of extended semen and allowed to stand for 60 s. A smear was prepared on a clean glass slide and examined under a 100X magnification microscope. Spermatozoa that were fully or partially stained were recorded as dead and those that were unstained were recorded as live. A total of 200 spermatozoa were counted, and the percentage was calculated.

### 2.4 Plasma membrane integrity

Plasma membrane integrity was evaluated using HOST. Briefly, 0.1 mL of semen was mixed with 1 mL of 150 mOsmol HOST solution in a glass tube and incubated for 60 min at 37°C (22). A phase contrast microscope was used to observe a drop of the incubated suspension at 40x magnification. A total of 200 sperm were counted. Spermatozoa with different forms of tail swelling were counted as sperm with an intact plasma membrane.

### 2.5 Acrosomal integrity

The percent intact acrosome was evaluated using the Giemsa staining method (23). On a glass slide, a smear was prepared using 20 μL of extended semen. It was air-dried and stained with Giemsa stain. A total of 200 numbers of spermatozoa were examined under 100X magnification of a microscope, and different forms of acrosomal damage were recorded. The percentage of spermatozoa with intact acrosomes was calculated and recorded.

### 2.6 Sperm DNA integrity

**Figure 1:**
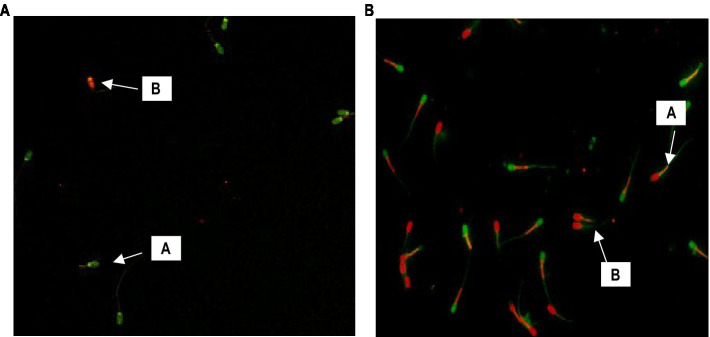
**(A)** Sperm with the arrow mark “A” emitting green fluorescence have intact DNA, whereas those with orange to red fluorescence are sperm with damaged DNA (400X). **(B)** Sperm with the arrow mark “A” emitting red fluorescence in the mid-piece region indicates high MMP, whereas those with green fluorescence are sperm with low MMP (400X).

The DNA integrity of the sperm cells was evaluated by the acridine orange (AO) staining technique (24). Thin smears were prepared on glass slides, air-dried, and fixed in Carnoy’s solution (one part glacial acetic acid to three parts methanol) for 2 h. Following fixation, smears were air-dried and stained for 5 min in the dark with freshly prepared AO stain (concentration: 0.19 mg/mL; TC262 HiMedia Labs), rinsed with distilled water, and then immediately examined with a fluorescent microscope (Nikon Eclipse T2i) using an excitation wavelength of 450–490 nm and a 530 nm barrier filter. Sperm cells with intact DNA fluoresced green and those with damaged DNA fluoresced from yellow-green to red (Figure 1).

### 2.7 Mitochondrial membrane potential

The MMPs were assessed using a cationic carbocyanine dye called JC-1(5,5′,6,6′-tetrachloro-1,1′,3,3′-tetraethyl benzimidazolyl carbocyanine iodide) (25). Briefly, 100 μL of semen sample was mixed with 1 μL of JC-1, counterstained with 4 μL of PI stock solutions, and incubated at 37°C in the dark for 30 min. Following incubation, sperm cells were spread on a grease-free glass slide and observed under a fluorescent microscope (Nikon Eclipse T2i) to count at least 200 cells. Those cells having mid-piece JC-1 aggregate-induced yellowish to orange fluorescence were considered to have high MMP levels, while those with green fluorescence were considered to have low MMP (Figure 1).

### 2.8 Artificial insemination

Semen was preserved in a BTS extender in 90-ml disposable plastic tubes for storage at 15°C. Each insemination dose (90 mL) contains at least 50 million spermatozoa per ml after warming to 37°C. The cervical insemination method was applied, and all the inseminations were carried out by the same technician. Sows in the second or third parity exhibiting natural estrus after weaning were maintained under standard housing, and management conditions were inseminated on the second and third days of oestrus. The sows were randomly distributed into four groups (control, T1, T2, T3, and T4). The control group received semen without any supplementation, while the T1, T2, and T3 groups received semen supplemented with 0.5, 1, and 2 μg ml^−1^ of Se-NPs, respectively. A total of 10 sows were inseminated from each of the groups. The boars and sows were randomly selected for insemination. For the insemination, semen preserved for up to 72 h was used.

### 2.9 Conception rate and litter size

The pregnancy was diagnosed using a veterinary ultrasound scanner (ExaGo Veterinary Scanner, IMV Technologies) after 25 days of gestation in sows. After farrowing, the fetuses born live, dead, mummified piglets, and total litter size were recorded.

### 2.10 Statistical analysis

The SPSS 20.0 statistical software was used to analyze the data. Data obtained in the present experiment were analyzed statistically for the main effect of experimental groups or hours of preservation using univariate analysis as per (26). Duncan’s new multiple range test (27) was used to test the significance of mean differences. The significance of different selenium nanoparticle-supplemented semen on conception rate was tested using a non-parametric chi-square (χ^2^) test. *p*-values < 0.05 were considered significant.

## 3 Results

The volume (ml), concentration (x 10^6^/ml), mass activity (0–5 grade), initial motility (%), live sperm (%), intact plasma membrane (%), and intact acrosome (%) recorded in the fresh semen were 238.50 ± 7.93, 173.25 ± 6.39, 4.15 ± 0.04, 90.72 ± 0.33, 87.27 ± 0.38, 65.12 ± 0.46, and 93.30 ± 0.29, respectively. The sperm quality attributes in different experimental groups at different hours of preservation are presented in Table 1.

**Table 1:** Evaluation of sperm quality attributes in different selenium nanoparticles supplemented and without supplemented groups at different hours of preservation.

Parameters	Hours	Control (without supplementation)	T1 (0.5 μL ml^−1^)	T2 (1.0 μL ml^−1^)	T3 (2.0 μL ml^−1^)	*p*-value
Total sperm motility (%)	0	87.4^A^ ± 0.45	88.37^A^ ± 0.47	88.47^A^ ± 0.46	87.75^A^ ± 0.43	0.340^NS^
	24	75.10^cB^ ± 0.57	77.30^bB^ ± 0.56	79.60^aB^ ± 0.47	75.52^cB^ ± 0.56	0.000**
	48	63.00^cC^ ± 0.64	67.25^bC^ ± 0.56	71.25^aC^ ± 0.55	64.00^cC^ ± 0.69	0.000**
	72	51.62^cD^ ± 0.60	56.75^bD^ ± 0.60	61.12^aD^ ± 0.52	53.25^cD^ ± 0.70	0.000**
	96	40.75^dE^ ± 0.52	45.75^bE^ ± 0.52	50.12^aE^ ± 0.48	42.75^cE^ ± 0.53	0.000**
	***p*-value**	0.000**	0.000**	0.000**	0.000**	
Live sperm (%)	0	86.12^A^ ± 0.41	86.72 ^A^ ± 0.37	87.05 ^A^ ± 0.28	86.22 ^A^ ± 0.43	0.279^NS^
	24	79.25 ^dB^ ± 0.40	81.40^bB^ ± 0.31	82.47^aB^ ± 0.28	80.22^cB^ ± 0.34	0.000**
	48	69.40^cC^ ± 0.56	72.17^bC^ ± 0.46	74.37^aC^ ± 0.42	70.52^cC^ ± 0.51	0.000**
	72	60.05^cD^ ± 0.71	62.95^bD^ ± 0.62	65.37^aD^ ± 0.58	61.57^bcD^ ± 0.60	0.000**
	96	50.57^cE^ ± 0.76	54.25^bE^ ± 0.64	56.82 ^aE^ ± 0.63	52.82^bE^ ± 0.68	0.000**
	***p*-value**	0.000**	0.000**	0.000**	0.000**	
Plasma membrane integrity (%)	0	63.60 ^A^ ± 0.46	63.92^A^ ± 0.52	64.62^A^ ± 0.47	63.70^A^ ± 0.49	0.451^NS^
	24	52.30^bB^ ± 0.53	53.57^abB^ ± 0.53	54.95^aB^ ± 0.55	52.70^bB^ ± 0.45	0.002**
	48	42.50^cC^ ± 0.61	44.37^bC^ ± 0.62	46.10^aC^ ± 0.61	43.25^bcC^ ± 0.57	0.000**
	72	33.92^cD^ ± 0.60	36.20^bD^ ± 0.65	38.07^aD^ ± 0.67	35.07^bcD^ ± 0.61	0.000**
	96	26.15^cE^ ± 0.57	28.32^bE^ ± 0.58	30.12^aE^ ± 0.61	27.15^bcE^ ± 0.57	0.000**
	***p*-value**	0.000**	0.000**	0.000**	0.000**	
Acrosomal integrity (%)	0	92.82^A^ ± 0.33	93.20^A^ ± 0.33	93.27^A^ ± 0.38	92.92^A^ ± 0.31	0.754^NS^
	24	82.85^cB^ ± 0.42	84.22^abB^ ± 0.38	85.15^aB^ ± 0.37	83.07^bcB^ ± 0.39	0.000**
	48	72.80^bC^ ± 0.51	74.95^aC^ ± 0.46	76.17^aC^ ± 0.42	73.65^bC^ ± 0.44	0.000**
	72	63.45^cD^ ± 0.64	65.87^abD^ ± 0.60	67.07^aD^ ± 0.62	64.60^bcD^ ± 0.62	0.000**
	96	53.75^cE^ ± 0.70	56.27^abE^ ± 0.66	57.92^aE^ ± 0.67	55.10^bcE^ ± 0.70	0.000**
	***p*-value**	0.000**	0.000**	0.000**	0.000**	
Sperm DNA integrity (%)	0	99.70^A^ ± 0.07	99.77^A^ ± 0.06	99.85^A^ ± 0.05	99.80^A^ ± 0.06	0.439^NS^
	24	99.60^AB^ ± 0.07	99.67 ^AB^ ± 0.07	99.75^A^ ± 0.06	99.70 ^AB^ ± 0.07	0.547^NS^
	48	99.40^bB^ ± 0.07	99.52 ^abB^ ± 0.07	99.70^aA^ ± 0.07	99.50^abB^ ± 0.08	0.057*
	72	98.95^cC^ ± 0.10	99.25 ^abC^ ± 0.08	99.42^aB^ ± 0.09	99.05^bcC^ ± 0.08	0.002**
	96	98.22^cD^ ± 0.12	98.80^aD^ ± 0.08	98.97^aC^ ± 0.06	98.50^bD^ ± 0.10	0.000**
	***p*-value**	0.000**	0.000**	0.000**	0.000**	
Mitochondrial membrane potential of sperm cell (%)	0	85.77^bA^ ± 0.30	86.25^abA^ ± 0.34	86.85^aA^ ± 0.29	85.75^bA^ ± 0.30	0.046*
	24	79.72 ^dB^ ± 0.23	81.52^bB^ ± 0.22	82.70^aB^ ± 0.16	80.35^cB^ ± 0.15	0.000**
	48	72.62^dC^ ± 0.29	74.77^bC^ ± 0.31	76.17^aC^ ± 0.21	73.65^cC^ ± 0.29	0.000**
	72	65.57^cD^ ± 0.29	67.50^bD^ ± 0.31	69.40^aD^ ± 0.28	66.67^bD^ ± 0.31	0.000**
	96	57.55^dE^ ± 0.31	60.07^bE^ ± 0.26	62.15^aE^ ± 0.27	59.17^cE^ ± 0.20	0.000**
	***p*-value**	0.000**	0.000**	0.000**	0.000**	

T1: Supplemented with 0.5 μg ml−1 of Se-NPs; T2: supplemented with 1 μg ml−1 of Se-NPs; T3: supplemented with 2 μg ml−1 of Se-NPs.

**p < 0.01; *p < 0.05; ^NS^Non-significant.

Means with different superscripts in a row (a, b, c, d) differed significantly.

Means with different superscripts in a column (A, B, C, D, E) differed significantly.

### 3.1 Sperm motility

The mean percentage of total motile sperm in the T1 and T2 groups was significantly (*p* < 0.01) higher as compared to control (C) at 24, 48, 72, and 96 h of preservation. At 96 h of preservation, significantly (*p* < 0.01) higher sperm progressive motility was observed in all treatment groups as compared to the control group. The ejaculates supplemented with 1 μg ml^−1^ of Se-NPs (T2) showed significantly (*p* < 0.01) higher sperm progressive motility as compared to the rest of the groups.

### 3.2 Live sperm

The mean percentage of live sperm also showed a similar trend as progressive motility, and it was observed that live spermatozoa were significantly (*p* < 0.01) higher in the T2 group (μg ml^−1^ Se-NPs) in comparison to other treatment and control groups at 24, 48, 72, and 96 h of preservation.

### 3.3 Plasma membrane integrity

The mean percentage of sperm with an intact plasma membrane was significantly (*p* < 0.01) higher in the T2 group as compared to other groups at 48, 72, and 96 h of preservation. No difference was observed between the T1 and T3 groups at any stage of preservation.

### 3.4 Acrosomal integrity

The mean percentage of sperm with intact acrosomes was significantly (p < 0.01) higher in the T1 and T2 groups in comparison to the control group at 24, 48, 72, and 96 h of preservation. No difference was observed the between T1 and T3 groups at any stage of preservation.

### 3.5 Sperm DNA integrity

The mean percentage of sperm with intact DNA was significantly (*p* < 0.05) higher in the T1 and T2 groups at 48, 72, and 96 h of preservation as compared to the control group, but there was no significant difference at 0 and 24 h of preservation.

### 3.6 Mitochondrial membrane potential of sperm cell

The mean percentage of sperm MMP was significantly (p < 0.05) higher at 0 h and 24, 48, 72, and 96 h (*p* < 0.01) of preservation in 1 μg ml^−1^ of Se-NPs supplemented group (T2) among all the groups.

### 3.7 Conception rate and litter size

The conception rate and litter size in the control and three treatment groups are presented in Table 2. The conception rate was significantly (p < 0.01) higher in T2 as compared to other treatment and control groups. The average litter size was observed to be higher in all treatment groups as compared to the control group, but the difference was non-significant. The average litter size at birth in control (C), 0.5 μg ml^−1^ (T1), 1 μg ml^−1^ (T2), and 2 μg ml^−1^ (T3) selenium nanoparticles supplemented groups was found to be 7.20 ± 0.35, 7.50 ± 0.42, 8.30 ± 0.55, and 7.30 ± 0.57, respectively.

**Table 2:** Conception rate and litter size in the different experimental groups.

Parameters	Control (Without supplementation)	T1 (0.5 μg ml^−1^)	T2 (1 μg ml^−1^)	T3 (2 μg ml^−1^)
Conception rate (%)	80^a^	90^a^	100^b^	90^a^
Litter size	7.20 ± 0.35	7.50 ± 0.42	8.30 ± 0.55	7.30 ± 0.57

Means bearing different superscripts in a row differed significantly (*p* < 0.01).

## 4 Discussion

The sperm membrane is put under a great deal of stress during preservation because of the generation of free radicals. It is generally known that raising ROS production slows down cell metabolism and makes sperm undergo an acrosome reaction. Although semen contains a variety of enzymatic and non-enzymatic antioxidants, their natural protection may not be enough to minimize the harmful effects of ROS. In such a situation, the antioxidant capabilities of semen can be increased by utilizing extenders enriched with exogenous antioxidants that either regulate, suppress, or block the oxidation process or prevent the generation of ROS in order to maintain sperm quality (17). Several NPs can be used in reproductive biological methods, particularly during semen preservation because of their antioxidant properties (18). Nevertheless, for the success of assisted reproductive outcomes, the potentially harmful effects of certain NPs should be taken into consideration. A study identified NPs as a driving force that triggers apoptosis and cell cycle arrest; therefore, it would be interesting to investigate any potential dose- and time-dependent harmful effects on the testes and male germ cells (28).

Reduced fertility may be due to severe injury to the sperm DNA, motility mechanism, plasma membrane, and acrosomal cap damaged during the processing of sperm. Oxidative stress causes lipid peroxidation in biomembranes leading to sperm abnormalities. The semen may be supplemented with different antioxidants that may act as free radical scavengers to protect the spermatozoa from reactive oxygen species (29). This antioxidant defense potential of semen is reduced during processing and preservation. Supplementation of antioxidants in the freezing diluents had a protective impact against lipid peroxidation, maintaining metabolic activity, and cellular viability (30, 31).

The present findings for sperm progressive motility, live sperm, plasma membrane integrity, and acrosomal integrity were in close agreement with the observations (32) and (33). They reported that nanoselenium supplementation of 1 μg/mL to the ram semen significantly (*p* < 0.01) improved sperm progressive motility, live sperm, plasma membrane integrity, and acrosome integrity compared to 2 μg/mL of nanoselenium and non-treated groups. Dorostkar et al. investigated the effect of *in vitro* supplementation of selenium on fresh and frozen semen of buffalo, and the results showed that the supplementation of 1 and 2 μg/mL of selenium in the extender significantly (*p* < 0.01) improved semen parameters (33). The present findings for sperm DNA integrity were in close agreement with the observations mentioned in Refs. (34) and (32). During the preservation phase, ROS production is more pronounced. The function of selenium in boosting antioxidant defenses, notably glutathione, which in turn controls excessive peroxide levels that might destabilize chromatin material, may explain why Se-NP-treated sperm samples had better DNA integrity. One of the primary techniques previously advised for evaluating sperm quality in human spermatozoa was the detection of MMP alterations (35). MMP, which is linked to intact mitochondria and may affect sperm motility, can therefore be used to detect the energetic condition of mitochondria. In addition, it was suggested that sperm with lower MMP were less likely to react to acrosomes (36). Such studies support the idea that NP supplementation (selenium) would be able to maintain the sperm MMP, giving insight into the sperm’s ability to fertilize. The present finding for the mitochondrial membrane potential of sperm cells was in close agreement with the observation (37), (38). The improvement of semen quality can be related to diminishing the effect of ROS by adding antioxidants, i.e., selenium nanoparticles, to the diluent. Kumaresan et al. reported that selenium is an important component of glutathione peroxidase, which, in turn, is important for preserving the structural integrity of the sperm membrane (39). According to the authors mentioned in Ref. (40), the addition of different levels of nano-selenium to the freezing medium was able to reduce free radicals and increase the motility and viability of post-thaw sperm. The present findings for conception rate and average litter size were higher than the observations (41). The sows used in the present study were Hampshire and *Niang Megha* (indigenous breed from India) crosses with 75% Hampshire inheritance. The litter size recorded in the study is typical for this crossbred. The conception rate and litter size were found to be higher in the treated selenium nanoparticle-treated groups. This indicates that the quality of the preserved boar semen improved after the addition of selenium nanoparticles to the extender that was used for insemination.

## 5 Conclusion

In conclusion, supplementation of Se-NPs in BTS extenders improves the semen quality of Hampshire crossbred boar spermatozoa during short-term preservation at 15˚C. The concentration of 1 μg ml^−1^ of Se-NPs resulted in the best semen quality and conception rate in comparison to other concentrations.

## Data availability statement

The original contributions presented in the study are included in the article/supplementary material, further inquiries can be directed to the corresponding author.

## Ethics statement

The animal study was approved by the Institution Animal Ethics Committee of The College of Veterinary Science & Animal Husbandry, Central Agricultural University, Aizawl, India.The study was conducted in accordence with local legislation and institutional requirements.

## Author contributions

DP: Investigation, Writing – original draft. DT: Investigation, Writing – review & editing. FA: Writing – original draft, Formal analysis. KL: Writing – original draft, Validation. GKa: Writing – original draft, Validation. TT: Formal analysis, Writing – original draft. HC: Writing – original draft, Investigation. RK: Formal analysis, Writing – original draft. GKh: Writing – original draft, Data curation. SD: Conceptualization, Supervision, Writing – review & editing.
